# Mini-med school for Aboriginal youth: experiential science outreach to tackle systemic barriers

**DOI:** 10.3402/meo.v20.29561

**Published:** 2015-12-23

**Authors:** Rita I. Henderson, Keri Williams, Lynden (Lindsay) Crowshoe

**Affiliations:** 1 Department of Family Medicine, Cumming School of Medicine, University of Calgary, Calgary, Canada; 2 Department of Community Health Sciences, Cumming School of Medicine, University of Calgary, Calgary, Canada

**Keywords:** Indigenous, youth, community engagement, outreach, science education, low-income, minority students

## Abstract

**Introduction:**

Addressing systemic barriers experienced by low-income and minority students to accessing medical school, the University of Calgary's Cumming School of Medicine has spearheaded a year-round, mini-med school outreach initiative for Aboriginal students.

**Method:**

Junior and senior high school youth generally attend the half-day program in classes or camps of 15–25, breaking into small groups for multisession activities. Undergraduate medical education students mentor the youth in stations offering experiential lessons in physical examination, reading x-rays, and anatomy. All resources from the medical school are offered in-kind, including a pizza lunch at midday, whereas community partners organize transportation for the attendees.

**Results:**

Opening the medical school and its resources to the community offers great benefits to resource-constrained schools often limited in terms of science education resources. The model is also an effort to address challenges among the medical professions around attracting and retaining students from underserved populations.

**Conclusion:**

The prospect of increasing admission rates and successful completion of medical education among students from marginalized communities poses a real, though difficult-to-measure, possibility of increasing the workforce most likely to return to and work in such challenging contexts. A mini-medical school for Aboriginal youth highlights mutual, long-term benefit for diverse partners, encouraging medical educators and community-based science educators to explore the possibilities for deepening partnerships in their own regions.

The half-day mini-medical school for Aboriginal youth of the University of Calgary's Cumming School of Medicine (CSM) came into being in 2008 to address under-representation of First Nations, Métis, and Inuit people within the realm of medicine (1). Requiring flexibility to accommodate the unique needs of on-reserve schools and community-based partners, the CSM's mini-med school for junior and senior high Aboriginal students is as much about making science content appealing and facilitation engagaing, as about making youth feel safe to attend higher studies at a distance from their communities. Lessons learned by organizers echo others (2) who call for a stepwise approach for Aboriginal youth outreach, to promote the medical professions and science education more broadly.

## Overcoming systemic barriers

Operated through the faculty's Aboriginal Health Program (AHP), the CSM's mini-medical school promotes institutional outreach goals and opportunities for strengthened partnerships between Aboriginal communities and postsecondary institutions. The AHP supports the CSM in achieving goals for improved health among Aboriginal people in Alberta, whereby the mini-med school falls under an objective to support the development of a pool of qualified Aboriginal applicants for medical training. Efforts to increase the representation of marginalized communities in the health professions emerge from recognition that a diversified workforce is more responsive to changing market demands (3), as the greatest predictor of a physician committing to practice in rural or underserved settings is their own background in such a community (4).

The Aboriginal population in the wider province of Alberta is diverse and its youth population is among the fastest growing demographic groups in Canada. Although Aboriginal persons compose 4.3% of the total Canadian population, Aboriginal children under 18 years of age compose 15.8% of Alberta's (5), representing First Nations, Métis, and Inuit peoples (also collectively referred to as FNMI peoples). Although the Canadian Constitution Act of 1982 refers to Aboriginal peoples as a collective group (6), differences between these are not merely cultural but are also historical and sociopolitical in nature. Within the CSM's region of Southern Alberta, there are five First Nations representing three cultural groups (i.e., Blackfoot, Tsuu T'ina/Dene, Stoney Nakoda) originating from the area and signatories of the 1877 Treaty 7, which settled their peoples on reservation territories. Métis peoples descend from 19th century groups of mixed Indigenous and Euro-American ancestry and are considered distinct from other Aboriginal peoples, whereas the Inuit are Indigenous to northern parts of the continent. The term Aboriginal itself has increasingly been critiqued in the Canadian context for generalizing and colonial undertones; nevertheless, many programs and organizations, such as the AHP, retain the terminology in their titles. This may soon change, as the federal government recently signaled a shift in preference for the term Indigenous.

As elsewhere in Canada, pathways into the city have intensified for young people from all of these groups; in urban centers, such as Calgary, nearly three-quarters of the Aboriginal population is among the first generation of their families to live in a city (7). Youth from rural Aboriginal communities face barriers to higher education because of distance from urban centers, particularly from cities where medical schools are based. Meanwhile, urban Aboriginal youth also face constraints that inhibit their studies, including reduced networks of social and financial support, concerns over safety and discrimination, and compromised access to quality K–12 education (8). Diverse Aboriginal persons also experience significant health inequities, displaying increasing rates of chronic disease and complications that lead to higher health care costs than among comparable non-Aboriginal groups (9, 10). Although negative health outcomes are consistently associated with social determinants of health (i.e., poverty (11, 12), discrimination (13), trauma (14)), reduced access to quality care further burdens this diverse population (15).

Members at the CSM have worked to redress such health inequities not only through the AHP but also through population health and intervention science research to tackle systemic barriers to well-being (16). However, until the 2015 release of the CSM's Strategic Plan for 2015–2020, which prioritizes greater community engagement through strengthening ‘relevance and connection with our local community’ (17), researchers and educators working to achieve Aboriginal health equity have found initiatives to be fragmented at best. A historical lack of system-level supports for integrating Aboriginal communities into health systems is reflected in efforts by CSM personnel to improve community outreach. For instance, prior to developing the mini-med school, AHP recruitment and admissions staff noted, at career fairs and presentations in area communities, that Aboriginal students and their mentors often insisted that the youth lack core skills to succeed in the field. Such perceptions suggest the internalization of systemic barriers to accessing professional training. Although low-income and minority youth in diverse settings frequently affirm that the profession is beyond reach (18), this does not necessarily mean that they are neither capable nor competitive. Arguably, such perceptions highlight the need for stronger premedical education and, in the absence of a large influx of resources and capacity to achieve this, more meaningful mentorship or career counseling integrated with a social and health equity approach. For medical schools, addressing such barriers requires inviting youth into the structures of the health system to render what have long been spaces of privilege and expertise familiar and approachable.

Models for medical school outreach to Aboriginal youth in Canada tend to involve summer science camps and community presentations encouraging potential applicants in high school (19). The CSM's mini-med school addresses two barriers that may evade such interventions. First, summer camps have the potential to be attended by self-selecting populations (i.e., youth with parental support and community infrastructure to link them to opportunities). The authors question whether the summer camp model therefore risks orienting prevailing outreach initiatives to youth already streamed toward science and postsecondary studies. Second, by the time the youth reach high school, many may already have lost motivation in the sciences, diminishing the range of young minds to which an admissions presentation could appeal. As both junior and senior high students can attend the mini-med school together, often attending multiple times over the years, the opportunity reinforces for a growing community of youth of distinct ages the idea of medicine as a viable and desirable career.

## Rolling-out a mini-med school with existing resources

**Fig. 1:**
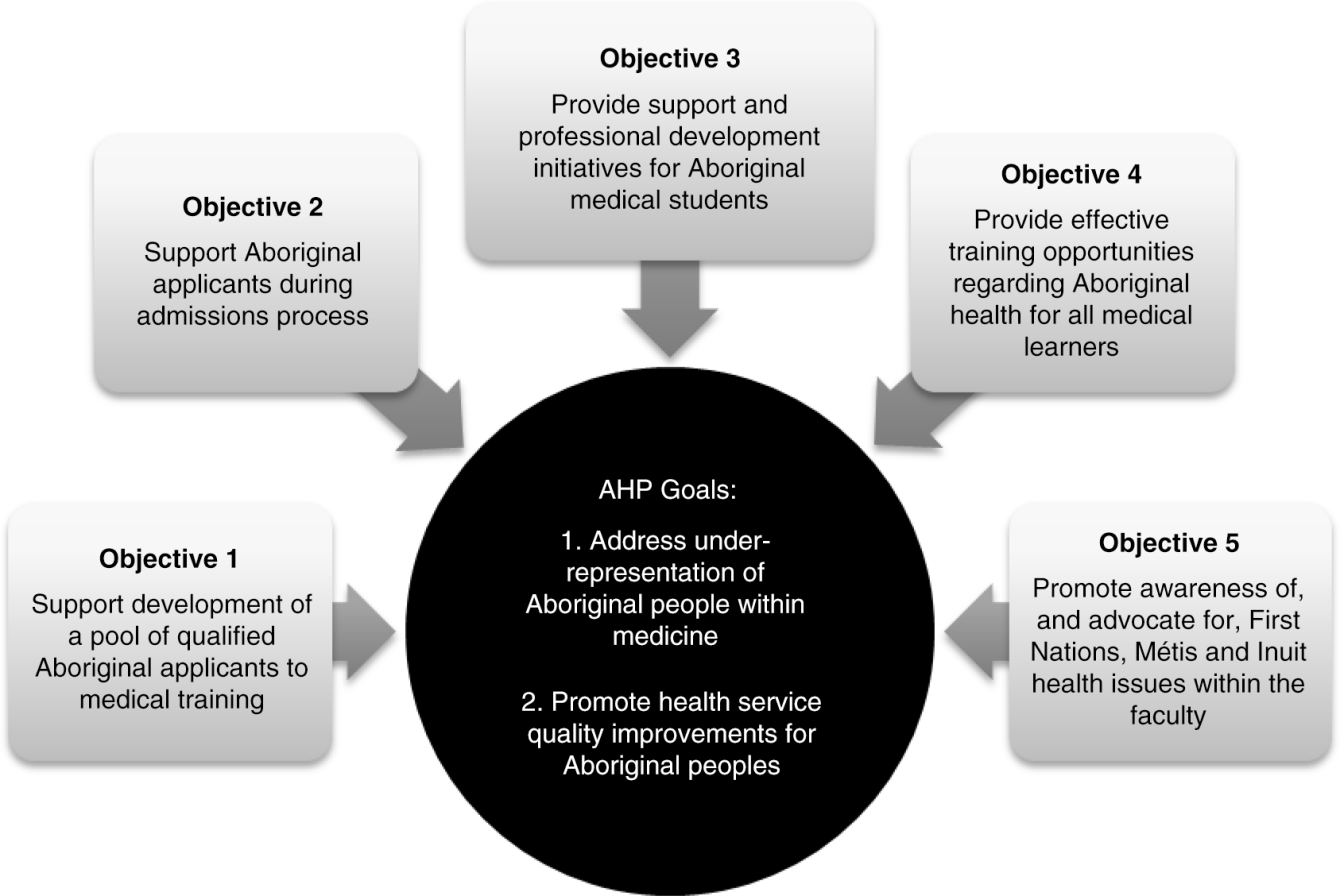
AHP commitments to improving Aboriginal peoples’ health.

In an effort to determine whether this is indeed a model that may increase youth engagement in the sciences in general, and Aboriginal student recruitment to medicine in particular, the AHP began a more deliberative process of pre/postevaluations in 2013 to gather data on youth experiences of the initiative. The remainder of this paper examines the mini-med school as a series of half-day educational activities aimed at improving health discipline career interest among junior and senior high school Aboriginal youth. Data from these pre/post participation surveys help us to formulate an increasingly more comprehensive approach to overcoming systemic barriers through distinct AHP initiatives, the mini-med school falling principally under Objective 1 to support the development of a pool of qualified Aboriginal applicants to medical training (Fig. 1). Such efforts belong to a larger task faced by health professional schools nationally, as the Truth and Reconciliation Commission of Canada recently called on the health professions to address barriers to recruitment and retention (20).

An initial question on attendees’ ‘interest in a career in medicine’ is now gathered upon youth arrival at the medical school, while six additional questions repeat at the end of their mini-med school experience the initial question and invite their thoughts on what the youth would like to know more about in terms of medicine, their rating of the quality of activities led by medical students, highlights of the day, areas for improvement, and other comments. Within a week of attending, qualitative exit interviews are attempted with mentors who accompany the youth groups either by phone or by sending an email inviting their reflections on the experience both for the youth and as a mentor. Four volunteer medical students were interviewed by the authors following the 10 August 2015 iteration (Table 1).

**Table 1:** Youth backgrounds for recent iterations

Date	Community/culture	Distance from CSM	Iterations/year
Feb 19, 2013	Morley (Stoney Nakoda FN)	62 km	
July 23, 2013	Multiple communities	n/a	2013
Sept 30, 2013	Tsuu T'ina FN (Dene)	Adjacent to city	4
Nov 20, 2013	Morley (Stoney Nakoda FN)	62 km	
June 4, 2014	Morley (Stoney Nakoda FN)	62 km	
July 14, 2014	Montana FN (Maskwacis Cree)	220 km	2014
Aug 6, 2014	Multiple communities	n/a	4
Nov 19, 2014	Tsuu T'ina (Dene)	Adjacent to city	
Apr 23, 2015	Siksika FN (Blackfoot)	80 km	
July 13, 2015	Montana FN (Maskwacis)	220 km	2015
Aug 10, 2015	Multiple communities	n/a	3

FN=First Nation.

### Youth profile

The half-day, small-group-based, multisession event is repeated three to four times per year on a schedule accommodating community partners. Classes of 15–25 junior high students from reserve communities attend during school months, whereas junior and senior high school students attend as part of summer science camps. Youth attendees from Treaties 6 and 7 territories (Central and Southern Alberta, respectively) represent Blackfoot, Stoney Nakoda, Dene, Cree, and Métis communities. During summer months, camp initiatives hosted more broadly at the University of Calgary plug into the opportunity, bringing students from even further afield, including remote areas of neighboring provinces of British Columbia and Saskatchewan, and tending to encompass a broader age range. In cases when students range from early junior high to nearing completion of high school, they are broken into groups with peers from their general age groups so that those closer to navigating the prospect of postsecondary studies may do so in the company of others facing such a life transition. Since 2013, youth attendees have arrived through such summer camps or from First Nation (reservation) communities. Mentors from the latter who accompany the youth have described variable resources for science education across Alberta First Nations, where underfunding means some communities lack relevant textbooks and lab equipment. For mentors from these First Nations, two of which have sent youth yearly since 2013, the mini-med school has been described as a valuable experiential learning opportunity.

### Format

The multisession event begins at 9:30 a.m. with a light breakfast and a brief introduction from an Aboriginal family physician on faculty whose presence is intended to model the approachability of the profession. The subsequent interactive sessions utilize simulations, demonstrations, presentations, and group problem-solving as skills development methods, mirroring medical education approaches at the CSM. These are broken into half-hour stations varying according to available resources; over the years, medical students have accumulated an abundance of activity plans that are stored in the AHP office and transmitted to each new set of volunteers to adopt or adapt. The AHP coordinator recruits five to eight student volunteers by sending out an email to a usually enthusiastic undergraduate medical education student body 2–3 weeks before a planned visit by the youth. This allows the mini-med school to operate on relatively short notice, keeping it flexible to partner timelines, while being restricted only by the ability to book space in the anatomy labs.

A physical exam station invites youth to experiment with stethoscopes, identifying their own bodies’ organs on diagrams. It also introduces youth to Harvey^®^ The Cardiopulmonary Patient Simulator (www.laerdal.com/ca/harvey), with which they practice locating pulses, listening to heart sounds, and identifying cardiac disease. A radiology station invites the youth to compete in a game of X-Ray/MRI/CT-Scan Jeopardy for candy rewards. An intubation station mentors learners through inserting a laryngoscope in dummies, and the anatomy station invites unique hands-on learning unavailable outside of a medical school. Whereas youth previously put a cast on a peer's arm, they now have the opportunity to build a cast on a balloon. In the words of one community mentor who recently accompanied a group, the ‘youth are exposed to totally novel experiences that they can't get anywhere else’ (Personal communication, Stoney Nakoda youth mentor, August 2015).

### Evaluation

Although a pre/postevaluation measures interest among attendees for postsecondary studies, it is early to assess the impact on attendees in terms of eventual admission into medical school specifically. Many initial participants in 2009–2010 will only recently be completing high school, and have yet to navigate undergraduate studies before they can even apply. Nevertheless, from at least three iterations for which comparable data exist, attendees consistently indicated increased interest in medicine immediately following the experience. It is worth noting that recent review of descriptive feedback in the postevaluation suggest that some youth may not have increased aspirations to pursue a career in medicine (the question posed) though they display increased interest in medicine in general, reflecting enhanced interest in health literacy. Interest was ranked on a 5-point Likert scale (1=not at all interested; 2=not very interested; 3=unsure; 4=a little interested; 5=very interested), such that the averages in Table 2 are out of a possible score of 5. Although overall interest consistently increased following the mini-med school experience, some youth nevertheless expressed decreased interest. Meanwhile, across the three iterations reviewed here, evaluation of the quality of activities led by medical students was strong, reflecting both positive assessment of the activities on offer and interactions with CSM personnel, who are primarily physicians-in-training.

**Table 2:** Interest in medicine immediately before/after half-day mini-med school session and activity evaluation

	Interest in medicine before session	Interest in medicine after session	Quality of activities led by medical students
June 4, 2014: Morley (Stoney Nakoda FN) (*n*=15)	3.75	4.13	4.5
July 13, 2015: Montana FN (Maskwacis) (*n*=17)	3.01	3.29	4.12
August 10, 2015: multiple communities attending university-wide science camp (*n*=17)	3.71	4.12	4.29

Youth feedback following the experience has noted a range of interests about what more they would like to learn. Some identified a desire to learn about medical school in general, whereas others keyed in on aspects of the human body they found curious (i.e., reflexes, lungs, skin, brain, nerves). Similarities in descriptive responses among youth who attended together suggest sessions that were particularly engaging for given iterations. For instance, this emerged in highlights from an 10 August 2015 session where 11 of 17 attendees identified the anatomy lab as a favorite experience (i.e., ‘dead bodies’, 'cadavers’, ‘poking dead bodies’, ‘human bodies donated after death’, etc.). Interestingly, descriptive feedback offers insight into more general interests in medicine, as youth identified desiring to learn more about how ‘to help a person in emergency’ and ‘ways to help a person’. Although youth routinely identify a range of subjects about which they would like to learn more (i.e., reflexes, pharmaceuticals, life at a medical school, x-rays, the brain, chemistry, personality, etc.), their postevaluation surveys indicate that these interests are shaped by interactions with medical student hosts who bring unique backgrounds and passion to interactions throughout the day. It nevertheless remains difficult to identify within the existing pre/postevaluation structure differences between populations of students who attend, as the inclusivity of the initiative tends to attract a seemingly diverse group (by age, sex, and interests) with each iteration.

Only recently, descriptive feedback from attendees began to indicate that this was not their first time attending such initiatives, for instance in claims that they like coming to the University of Calgary whenever possible and desiring a more comprehensive tour of different real-life areas of the medical school. In the summer of 2015, organizers verbally confirmed with at least five youth that they had attended previously. This led to the recent addition to the evaluation survey of a question about whether this is a first or repeat experience of the mini-medical school and other on-campus outreach initiatives, enabling organizers to follow-up with mentors in exit interviews with more specific questions about whether repeated attendance improves youth interest in science, academic performance, or promotion of attending the mini-medical school among peers.

As the initiative has developed, organizers increasingly aim for youth to experience mini-med school in junior high with someone following up when the youth are in high school by visiting communities to help students anticipate the logistics of further studies (i.e., how to apply, pay for, and otherwise navigate undergraduate careers). As one medical student volunteer put it, the mini-med school ‘helps to lay out a roadmap for how to even get into med school, but there are so many other variables between here and getting there’, (Personal communication, 2nd year medical student, August 2015). The challenge ahead is to establish enough continuity with community partnerships to develop longitudinal tracking of attendees, not only to follow their possible pathways into postsecondary studies but also to provide additional resources for overcoming the barriers that undermine this eventuality.

### Costs and constraints

Relying on existing resources, the mini-med school draws on the volunteer enthusiasm of mainly second-year medical students in a position to fit a half-day of outreach into their preclerkship schedules. Among a number of projects falling under the umbrella of the AHP coordinator's portfolio, the coordinator takes charge of scheduling two volunteers per half-hour session, arranging lunch for attendees, and booking rooms. The volunteers’ and coordinator's time, as well as the pizza lunch, are in-kind contributions by the CSM, whereas community partners take charge of transportation.

Community partners also prepare the youth for the anatomy lab, securing prior written consent from guardians and ensuring that pants and closed-toe shoes are worn. At times, community-based schools have become overwhelmed by one community challenge or another, making a planned attendance fall through. Teacher burnout in reserve communities has also challenged the AHP's coordinator to continuously reestablish relationships with teachers and administrators. In these contexts, the mini-med school offers an added support to resource-constrained schools for nurturing science education capacity and confidence among youth.

## Discussion: mini-med school for community engagement

Mini-medical schools can increase health literacy among target populations (21) and inform prospective student expectations of the profession to enhance the quality of the applicant pool (22). Oriented by a social accountability framework, the AHP's initiative reinforces that a medical school's scope of community engagement should reach well beyond community health service (23). As such, it enables the CSM to invest significantly upstream in the potential applicant pool by interfacing with youth across the age spectrum, rather than targeting only those close to university age. As the initiative expands the medical school's scope of community engagement, relationships are with schools, camp coordinators, science education networks, social services, businesses, as well as both traditional and formal community leadership, not just with conventional health services within Aboriginal communities.

New partnership horizons pose new challenges and opportunities for developing metrics that might validly reflect programmatic impact. Although existing pre-/postevaluations address immediate impacts on interest in pursuing a medical career, longer-term impacts depend on the ability to forge sustained relationships with partnered organizations in order to endure staff turnover. In recent years, this has been possible with three different First Nations whose youth have attended multiple times, as well as a summer camp that has returned twice. Initial efforts are currently underway to establish in collaboration with community-based organizers, a tracking tool to report to the medical school the number of former attendees who go on to pursue postsecondary education, as well as the proportion of these entering the sciences (i.e., biology, chemistry) and related professions at the undergraduate or technical school levels (i.e., engineering, nursing, emergency medical technician).

Given the focus on reducing barriers and in light of the diversity of the Aboriginal population within the wider region (i.e., urban/rural; multiple cultural groups), tracking attendee interest in the sciences across time is more reliable than comparing their interest to that among a generic Aboriginal youth population within the region. Although the initiative targets a relatively distal outcome by addressing increased access to the health professions, the potential for monitoring the incremental effect of the program on youth interest is expected to be enhanced through more systematic, community-based outreach to high school students, focusing on supporting these to navigate postsecondary admissions processes, undergraduate program selection, and transitions into higher studies. With the CSM's 2015 launch of an Indigenous Health Dialogue process to engage area communities in deepened partnerships with teaching, research, and service branches of the school, forging longitudinal relationships with former attendees appears increasingly possible, thanks in large part to growing support for the mini-med school program within the CSM. With the recent integration of the mini-medical school's organizers (Henderson; Crowshoe) into leadership roles in the CSM's Office for Strategic Priorities and Community Engagement, the initiative has acquired greater human resource capacity and potential for follow-up outreach. In turn, this is expected to enable yearly implementation of the pre/postevaluation survey as a baseline with non-attendee youth of the same age range from the same communities or schools as attendees. Given the diversity of the Aboriginal population in Southern Alberta, baseline data would only be compared with those students from the same communities or schools.

Challenges emerge from strong reliance on community-based educators and youth development workers to keep in touch and articulate their needs to medical school partners. Since increasing the initiative's recurrence to three to four iterations a year in 2012, organizers have noted the high turnover of community-based contacts. This has required more effort by the AHP coordinator to send out frequent reminders of the opportunity in order to enhance the profile of the initiative within communities and schools more broadly. Lack of continuity in community-based contacts also complicates the intention of organizers to establish follow-up outreach within communities to support high school youth in preparing for postsecondary studies. Fostering greater continuity in partnerships will require increased human resources investment on the medical school's side to strengthen community connections and ensure greater responsiveness to community needs.

The impact of a mini-med school model is not restricted to increasing minority students who meet the requirements for medical school admission. The community-based partnerships themselves offer potential for establishing the necessary groundwork to sustain support for youth from underserviced communities to navigate the many stages behind reaching admission to health professional programs in the first place. This is achieved through experiential learning opportunities to support science education for youth located in resource-constrained contexts, where teacher burnout and limited resources undermine exposure to potentially fulfilling career opportunities. The flexibility to community needs embedded in what is a long-term pipeline approach for recruitment models a low-cost initiative that may be advocated by school boards and other community organizations to strengthen partnerships with medical and other health professional schools. Although low-income and minority students are targeted, youth science education is equally enriched through experiential learning. Therefore, although this article outlines how one medical school makes such learning opportunities possible, it is important to note the crucial role played on the one hand by community organizations and educators in seeking such opportunities for youth, and on the other hand by medical educators who recognize advantages for the health professions more broadly.
